# Hidden Treasures: Macrophage Long Non-Coding RNAs in Lung Cancer Progression

**DOI:** 10.3390/cancers13164127

**Published:** 2021-08-17

**Authors:** Annika Karger, Rajender Nandigama, Albrecht Stenzinger, Friedrich Grimminger, Soni Savai Pullamsetti, Werner Seeger, Rajkumar Savai

**Affiliations:** 1 Max-Planck Institute for Heart and Lung Research, Member of the German Center for Lung Research (DZL), Member of the Cardio-Pulmonary Institute (CPI), Parkstr. 1, 61231 Bad Nauheim, Germany; annika.karger@mpi-bn.mpg.de (A.K.); Rajender.Nandigama@mpi-bn.mpg.de (R.N.); soni.pullamsetti@mpi-bn.mpg.de (S.S.P.); werner.seeger@innere.med.uni-giessen.de (W.S.); 2 Institute of Pathology, University Hospital Heidelberg, 69126 Heidelberg, Germany; albrecht.stenzinger@med.uni-heidelberg.de; 3 Institute for Lung Health (ILH), Justus Liebig University, 35392 Giessen, Germany; friedrich.grimminger@innere.med.uni-giessen.de; 4 Department of Internal Medicine, Member of the DZL, Member of CPI, Justus-Liebig University Giessen, 35392 Giessen, Germany; 5 Frankfurt Cancer Institute (FCI), Goethe University, 60596 Frankfurt am Main, Germany

**Keywords:** lncRNA, macrophage, TAM, lung cancer, tumor microenvironment

## Abstract

**Simple Summary:**

Cancer immunotherapy mainly targets immune system components, such as immune-suppressive networks generated by cancer cells in the tumor microenvironment (TME). Programmed cell death ligand 1, which is a secretory immune-suppressive factor, is released by tumor-associated macrophages (TAMs). The TME also disrupts production of tumor-specific T cells and generates immunosuppressive leukocytes, regulatory T cells, and myeloid-derived suppressor cells. Immune checkpoint inhibitors are effective in various cancers but only in a subset of patients. Non-coding RNAs, such as microRNAs (miRNAs), long non-coding RNAs (lncRNAs), and circular RNAs (circRNAs), are dysregulated in cancer through transcriptional, post-transcriptional, and epigenetic changes and have significant roles in cancer initiation and progression, which depends on deregulation of lncRNA expression. TAM function can be influenced by lncRNAs in various ways. However, our understanding of lncRNA dysregulation and function in cancer remains in the early stage.

**Abstract:**

Ever since RNA sequencing of whole genomes and transcriptomes became available, numerous RNA transcripts without having the classic function of encoding proteins have been discovered. Long non-coding RNAs (lncRNAs) with a length greater than 200 nucleotides were considered as “junk” in the beginning, but it has increasingly become clear that lncRNAs have crucial roles in regulating a variety of cellular mechanisms and are often deregulated in several diseases, such as cancer. Lung cancer is the leading cause of cancer-related deaths and has a survival rate of less than 10%. Immune cells infiltrating the tumor microenvironment (TME) have been shown to have a great effect on tumor development with macrophages being the major cell type within the TME. Macrophages can inherit an inflammatory M1 or an anti-inflammatory M2 phenotype. Tumor-associated macrophages, which are predominantly polarized to M2, favor tumor growth, angiogenesis, and metastasis. In this review, we aimed to describe the complex roles and functions of lncRNAs in macrophages and their influence on lung cancer development and progression through the TME.

## 1. Lung Cancer

Lung cancer is one of the most frequent and most deadly types of solid cancers worldwide, and a highly complex, very heterogeneous disease. Histological classification divides lung cancers into non-small cell lung carcinoma (NSCLC, approximately 85% of the cases) and small cell lung carcinoma (SCLC, approximately 15% of the cases). NSCLC can be classified further into adenocarcinoma, squamous cell carcinoma, and large cell carcinoma. Although lung cancer is treatable with early-stage radical interventions, it remains challenging because >70% of patients relapse and expire [1,2,3,4]. In addition, the broad use of cytotoxic chemotherapies in lung cancer has reached a plateau [5]. In the past decade, exploring cancer treatment options has led to alternative therapies, such as immunotherapies and targeted therapies [6]. Cancer immunotherapies mainly target particular immune system components, such as immune-suppressive networks in the tumor microenvironment (TME), to enhance antitumor activity. Cancer cells shape their microenvironment and generate immune-suppressive networks, which finally overwhelm immunity and tumor progression [7]. Tumor cells and stromal cells, such as tumor-associated macrophages (TAMs), create an immunosuppressive environment by secreting immune-suppressive factors, such as programmed cell death ligand (PDL) 1 [8]. Additionally, the TME disturbs production of tumor-specific T cells like cytotoxic T cells (CTLs), and generates immunosuppressive leukocytes, regulatory T cells (Tregs), and myeloid-derived suppressor cells [9,10]. Various immune checkpoint inhibitors, such as CTL-associated protein 4 (CTL-4) and programmed cell death 1 (PD1), have been developed in immune-based therapies in different types of cancer [11,12]. Although immune checkpoint inhibitors provide durable responses to various cancers, they are effective in only a subset of patients [13]. Furthermore, recent advances in cancer research have led to molecular targeted therapies targeting identified gene mutations and molecular alterations to treat cancer [14]. Genomic profiling with advanced techniques like next-generation sequencing has identified molecular alterations and driver mutations in lung cancer [15]. The majority of genetic aberrations are in the epidermal growth factor receptor (EGFR), Kirsten rat sarcoma viral (KRAS), tyrosine-protein kinase MET (MET), anaplastic lymphoma (ALK), PI3KCA, ERBB2, and BRAF. EGFR and KRAS mutations are the most frequently found mutations in lung cancer. These specific mutations support tumor growth and proliferation by activating several signaling pathways [16]. These findings recently led to the development of targeted agents like T tyrosine kinase inhibitors (TKI) to target EGFR-activating mutation and to use targeted-based therapies in lung cancer. However, only about 20% of patients benefit from these targeted therapies in lung cancer patients with drug-sensitive mutations. Furthermore, drug resistance caused by genetic alterations is a major impediment to long-term therapeutic outcomes [17,18]. As a result, more in-depth research is required to develop new cancer-targeted medicines.

Non-coding RNAs, such as microRNAs (miRNAs), long non-coding RNAs (lncRNAs), and circular RNAs (circRNAs), are commonly dysregulated in cancer through the transcriptional, post-transcriptional, and epigenetic changes and have been shown to have significant roles in cancer initiation and progression [19,20]. Deep sequencing and microarray profiling investigations have further shown that deregulation of lncRNA expression is also a critical factor in initiation and progression of lung cancer [21,22]. Some recent studies also found that lncRNAs can influence TAM function in various ways [23,24,25]. However, understanding lncRNA dysregulation and function in cancer is still in its early stages. In this present review, we will first briefly discuss activation and polarization of TAMs and their role in lung cancer progression, and then specially focus on lncRNAs’ functions and mechanisms, influence of lncRNAs on TAMs, their roles in TME inflammation, and regulation pathways in lung cancer. Finally, we will discuss the therapeutic potential of lncRNAs in diseases, particularly lung cancer.

## 2. Tumor Microenvironment and Tumor-Associated Macrophages

Extracellular matrix (ECM), blood and lymphatic vasculature, stroma, and cells of the immune system that may be residing in the affected tissue or invaded from the periphery comprise the tumor microenvironment (TME). Infiltration of immune cells in the TME is highly associated with the clinical outcome of cancer patients [26,27,28]. Cells of the adaptive immune system, such as lymphocytes, and cells of innate immunity, such as macrophages, neutrophils, eosinophils, mast cells, dendritic cells, and natural killer cells, are among the infiltrating immune cells in TME. A growing number of reports have indicated that cross-talk between tumor cells and immune cells in the TME and production and secretion of cytokines by immune cells regulate tumor initiation, progression, and metastasis [29,30].

Macrophages are versatile immune cells that provide innate immune responses against invading pathogens and also play a role in maintaining tissue homeostasis. Macrophages that infiltrate into the tumors in the TME are known as tumor-associated macrophages (TAMs). Recent research developments have shown that TAMs affect tumor progression, metastasis, angiogenesis, and immunosuppression [31].

Macrophage cross-talk with tumor and stromal cells in the TME changes macrophage signaling and epigenetic and metabolic mechanisms to form different functional TAM phenotypes, such as the classical subtype of activated macrophage M1 or alternative subtypes of activated macrophage M2. Conventional phenotyping distinguishes macrophage subpopulations as M1, which express a high level of MHC class I and class II proteins, possessing proinflammatory and antitumor activities; from activated, alternative M2, which is mainly involved in suppressing inflammation and promoting tumor growth [32]. These two major phenotypes are distinct in expression markers, metabolic characteristics, and gene expression profiles. M1 macrophages arise in inflammatory settings induced by type 1 T helper (Th1) cytokines such as interferon-gamma (IFNɣ), Toll-like receptor agonists like lipopolysaccharide (LPS), and granulocyte–monocyte colony-stimulating factor (GM-CSF) [33]. M1 macrophages show efficient antigen presentation, and are characterized by high interleukin (IL)-12 and IL-23, low IL-10, and high levels of inducible nitric oxide synthase (iNOS). M2 macrophages are anti-inflammatory phenotypes found in environments associated with type 2 T helper (Th2) cytokines. M2 macrophages are classified into four different subtypes such as M2a (IL-4, IL-13), M2b (immune complexes and IL-1), M2c (IL-10, glucocorticoids, and transforming growth factor β), and M2d (IL6, leukemia inhibitory factor). M2 macrophages are characterized by low IL-12 and IL-23, high IL-10, and high arginase 1 (Arg1) [34,35,36]. Although M2 macrophage phenotype subtypes have common anti-inflammatory and immunoregulatory functions, these subtypes also play a different role in different diseases [37].

### 2.1. Activation and Polarization of TAMs

**Figure 1:**
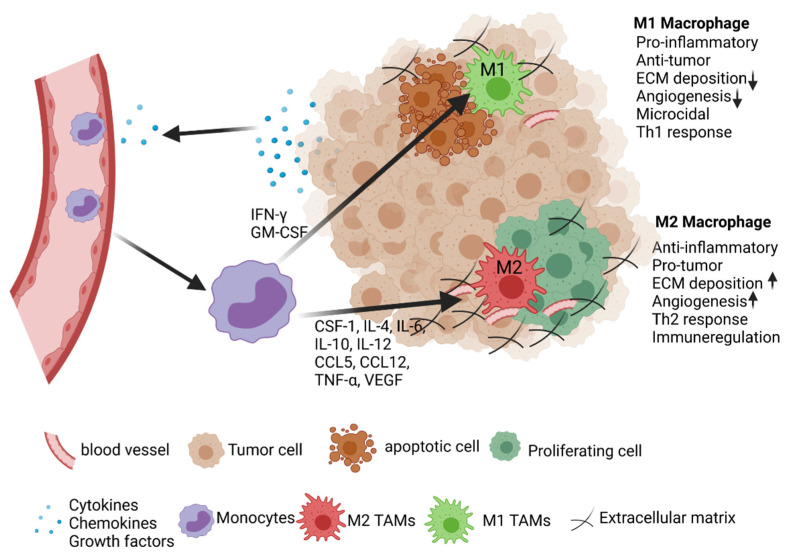
TAMs activation and polarization in the TME. Various chemokines and growth factors released by stromal and tumor cells in the TME activate and recruit monocytes and activate macrophages either to a protumor M2 phenotype (CSF1, IL-4, IL-6, IL-10, IL-12, CCL5, CCL12, TNF-α, VEGF, Lactate) or antitumor M1 phenotype (GM-CSF, IFNγ). Further, by interacting and releasing secretory factors, M1 macrophages are able to induce tumor cell apoptosis and a Th1 response, whereas M2 macrophages induce tumor cell proliferation, angiogenesis, and lead to Th2 response and immune regulation.

Macrophage polarization is a highly plastic process through which macrophages differentiate into specific phenotypes with functional differences in response to local microenvironment signals. In recent years, significant progress has been made in understanding macrophage polarization and the molecular mechanisms in various cancer types (Figure 1) [38,39]. Furthermore, Th1 cytokines (IFNɣ, TNF-α) drive macrophages to form the M1 phenotype, and Th2 cytokines (IL-4, IL-13) induce macrophages to form the M2 phenotype [38]. Several studies found that accumulation of M2-TAMs in the TME correlates with advanced tumor progression and poor prognosis of cancer, such as breast cancer [40,41,42]. These observations suggest that TAMs are mainly M2 phenotype macrophages.

Cytokines, GM-CSF (CSF2), and macrophage colony stimulating factor (MCSF, also CSF-1) are known to regulate macrophage activation and differentiation. GM-CSF is mainly involved in M1 phenotype polarization, whereas CSF1 is associated with M2 phenotype polarization [43]. CSF-1, which is highly expressed in many tumors, induces M2 polarization. CSF1 binds to CSF-1R and activates upregulation of PLCᵧ2, STAT3, and ERK1/2 [44]. C-C motif ligand 2 (CCL2) is expressed in many tumors, and can polarize macrophages toward the M2 phenotype. Furthermore, in CCL2-mediated macrophages polarization occurs via C- C chemokine receptor 2 (CCR2) expressed on macrophages. In vivo studies in tumor-bearing CCR2 knock-out mice and CSF-1 depleted tumor graft models have shown reduced TAM density, inhibition of protumor cytokine expression, and prolonged survival in these mice [45,46]. Furthermore, in vitro co-culture models have shown that tumor cell-macrophage co-culture induced M2-like polarization via increased expression of IL-10, IL-12, IL-6, TNF-α, CCL5, CCL22, and CSF1 [47]. Another study showed that IL-6 via STAT6 signaling activated macrophage polarization to the IL-4 dependent M2 phenotype by overexpressing IL-4 receptor [48]. IFNɣ has been identified as a potent agent for modulating macrophage polarization [49]. Furthermore, an in vivo study in IFNɣ knock-out mice showed the polarization of macrophages to the M2 phenotype and increased tumor growth [50].

Hypoxia occurs quite frequently in solid tumors and helps modulate the TAM phenotype in the TME. Migration of TAMs into the hypoxic area is induced by hypoxia-mediated chemokines, such as CSF1, CCL2, CCL5, VEGF, semaphorin 3A, endothelin, stromal cell-derived factor 1α, eotaxin, oncostatin M, and endothelial cell monocyte-activating polypeptide II (EMAP-II) [51,52,53,54]. Furthermore, metabolite lactate, one of the key inducers of M2 polarization, is produced in oxygen-deprived areas by tumor cells [55].

### 2.2. Role of TAMs in Lung Cancer

TAMs are one of the most abundant immune cells in the TME of lung cancer. It has also been reported that TAMs contribute to lung cancer progression, angiogenesis, invasion, metastasis, immunosuppression, and resistance to chemotherapy by releasing cytokines, growth factors, and chemokines [56]. Studies in lung cancer have shown that TAMs express M1 markers during tumor formation and switch to the M2 phenotype during lung cancer progression [57,58]. Several studies have found that having a high density of M2 phenotype TAMs in the TME correlated with poor survival in lung cancer [59,60,61]. Furthermore, a spatial density and distribution study showed that M2 TAM predominance, a lower density of M1 TAMs in the tumor center, and high proximity of tumor cells to M2 TAMs in the invasive margin were linked to poor prognosis in NSCLC [62].

In NSCLC, cancer stemness is promoted by M2 macrophages secreting IL-10 via the JAK/STAT1/NF-κB/Notch1 signaling pathway [63]. Furthermore, a clinical study reported that high IL-10 expression in TAMs plays a significant role in the tumor progression, invasion, metastasis, and poor prognosis of NSCLC [64]. Additionally, TAMs with high CSF-1R expression have been associated with poor prognosis in lung cancer patients [65]. In vitro co-culture studies in THP1 cells with NSCLC A549 or H1299 cells have shown high IL-6 expression in THP1 cells, which enhances the invasive ability of cancer cells by regulating EMT [66]. A blockade of IL-6 expression in TAMs inhibited invasion and angiogenesis in lung cancer [67]. IL-8 upregulation in tumors by TAMs showed an increase in tumor angiogenesis and poor patient survival in NSCLC [68]. TGF-β secreted by TAMs upregulates SOX9 expression, which promotes EMT and enhances tumor cell proliferation, migration, and invasion in lung cancer via the TGF-β/SOX9 axis [69]. In vivo study in lung adenocarcinoma A549 cells showed that TAMs promoted proliferation, invasion, and migration by activating the PI3K/AKT signaling pathway [70]. MMPs, such as MMP-9 and MMP-2 secreted by TAMs, degrade the ECM and induce lung cancer invasion [71]. Recent studies have reported that TAMs secrete chemokines, such as CCL5, CCL8, CCL18, CCL22, and MIP-3α, that may play a significant role in lung cancer [72,73,74]. In vivo studies in Lewis lung carcinoma (LLC) mouse model studies using genetic ablation of monocyte recruitment markers CCR2 and CX3CR1 and macrophage depletion using clodronate also have shown inhibited tumor growth and metastasis [75]. Recent studies also reported that transcriptome analysis in TAMs showed upregulation of Wnt/β-catenin signaling proteins in lung cancer. Furthermore, high expression of β-catenin and FOS-like antigen 2 correlated with poor prognosis in lung cancer [76]. In addition, TAMs also promote angiogenesis in lung cancer by secreting pro-angiogenic factors, including VEGF. A high number of TAMs in tumors is associated with intra-tumoral vessel counts in NSCLC [77].

TAMs also play a significant role in the immunosuppression and immunoregulatory functions of other cells. Expression of programmed cell death 1 (PD1) in TAMs inhibits phagocytosis and immunity [8,78]. Furthermore, it has been reported that TAMs limit the efficacy of PD-1 treatment by blocking CD8 cells that reach to tumor cells. In addition, alveolar macrophages (AM) contribute to the pre-metastatic niche by suppressing CD8 T cell responses in the lungs. Additionally, upregulation of PD-L1 expression in TAMs by IL-10 induces the immunosuppression of T cells [79], and CCL22 secreted by TAMs in the TME promotes immunosuppression by recruiting T-regs [80]. It has also been reported that TAMs accumulate inside or adjacent to tumors following chemotherapy. In vivo studies in LLC1 tumor models have shown that chemotherapy-induced release of CXCL12 from neoplastic cells enhanced infiltration of TAMs that contributed to tumor relapse [81].

## 3. Long Non-Coding RNAs

The ability to sequence whole genomes and transcriptomes identified all kinds of transcripts and showed that approximately 90% of the human genome is actively transcribed. However, only about 2% of those transcripts are translated into proteins, with the rest remaining as non-coding RNAs [82,83]. The most prominent non-coding RNAs are microRNAs (miRNAs) and long non-coding RNAs (lncRNAs). MiRNAs are short, single-stranded RNA sequences (21–24 nucleotides) and mainly function via binding mRNAs, leading to their degradation and therefore translational inhibition. On the other hand, the large group of lncRNAs that comprise all ncRNAs > 200 nucleotides long are much more heterogeneous because they form complex secondary and tertiary structures and interact with proteins, DNA, or other RNAs.

### 3.1. Functions and Mechanisms

**Figure 2:**
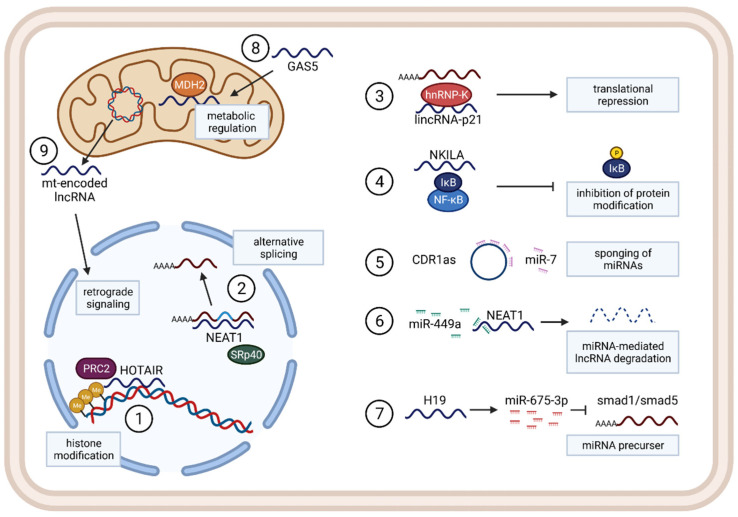
Representative examples for general functions and mechanisms in lncRNA regulations in different cellular compartments. 1: lncRNA HOTAIR regulating histone modification by binding the PRC2 complex, recruiting it to specific genomic regions. 2: NEAT1 scaffolding SRp40 together with mRNAs, leading to regulation of alternative splicing. 3: cytoplasmatic lincRNA-p21 functions as a translational repressor, binding hnRNP-K together with a mRNA. 4: lncRNA NKILA binding the NF-κB complex inhibits phosphorylation of IkB, leading to loss of NF-κB activation. 5: CircRNA CDR1as contains miR-7 target sequences, functioning as a miRNA sponge. 6: lncRNA NEAT1 is degraded after miR-449a binding. 7: lncRNA H19 serves as a precursor RNA for several miRNAs, such as miR-675-3p that inhibits smad1 and smad5. 8: nuclear-encoded lncRNA GAS5 is able to enter the mitochondria, binding MDH2 protein and influencing TCA flux and cellular metabolism. 9: mitochondrial genome-encoded lncRNAs can traffic between the mitochondria and nucleus and provide retrograde signaling functionally.

As a large and diverse group of regulatory non-coding RNAs, lncRNAs seem to be poorly evolutionarily conserved between species and often show tissue and cell-type-specific expression patterns. They are known to regulate through various mechanisms, for example, functioning as a guide for target proteins such as transcription factors to specific regions in the genome or by building a scaffold, binding different proteins, and bringing them closer together. Additionally, lncRNAs can be localized in different compartments of the cell and regulate on the transcriptional level, by alternative splicing, by regulating translation, or by directly interacting with proteins to influence their modification and activation (Figure 2).

Nuclear lncRNAs are known to regulate gene expression: e.g., through chromatin modification, such as the HOX antisense intergenic RNA (HOTAIR). HOTAIR is highly expressed in NSCLC tissue and recruits the PRC2 complex, leading to histone methylation and transcriptional repression [84]. Nuclear paraspeckle assembly transcript 1 (NEAT1), on the other hand, is localized in nuclear speckles, and associates with SRp40, affecting alternative splicing of transcripts (Figure 2) [85].

Cytoplasmatic RNAs can affect gene expression on the post-transcriptional level, such as lncRNA 1/2-sbs-RNA binding to BACE1-AS-mRNA, increasing its stability and enhancing expression or such as lincRNA-p21, binding to a target mRNA sequence, and enhancing interaction with the post-transcriptional repressor RCK and FMRP, consequently inhibiting translation [86]. Other lncRNAs can interact with proteins and influence protein modification, such as lncRNA NKILA, binding the NF-κB complex and repressing phosphorylation of IκB, thus inhibiting NF-κB activation [87]. Additionally, lncRNAs and miRNAs can influence each other due to their possible sequence complementarity, and lncRNAs can contain binding sequences and function as miRNA sponges, serving as endogenous competitors for miRNA–mRNA binding, thereby interfering with the miRNA-targeted degradation of a specific mRNA [88]. As endogenous competitors, LncRNAs can have the form of a circRNA, a newly identified large class of RNA that is predominantly localized in the cytoplasm of cells. CDR1as, for instance, is a circRNA containing 74 binding sequences for miR-7 [89]. On the other hand, miRNAs can regulate the stability of lncRNAs by binding to them and causing degradation, such as in the case of miR-449a in lung cancer, which binds to NEAT1 to inhibit the lncRNA-function [88]. It has also been found that lncRNA transcripts can serve as precursors for miRNAs, such as lncRNA H19, which gives rise to miR-675-5p and miR-675-3p that inhibit smad1 and smad5, among others (Figure 2) [90].

More recently, several mitochondrial lncRNAs were identified as regulators of cellular metabolism. Some examples are nuclear-encoded lncRNA growth arrest specific 5 (GAS5) that enters the mitochondria, binds to MDH2, and regulates TCA flux under stress conditions [91], or even mitochondrial genome-encoded lncRNAs, which are mostly described as sending retrograde signals to the nucleus (Figure 2) [92].

### 3.2. LncRNAs in Cancer

Given the abilities of lncRNAs for controlling all kinds of processes within the cell, it is not surprising that disruption can lead to aberrant gene expression and is associated with multiple diseases, especially cancer. In previous years, more lncRNAs have been found to regulate the occurrence and progression of many aspects of tumors by targeting genomic mutations, DNA damage, metabolic disorders, and EMT or cancer cell stemness.

Accumulation of DNA damage plays an important role in cancer development. By regulating proteins that are involved in DNA damage repair or stress response, lncRNAs can influence the mutational burden of cells. Some examples are lncRNA MEG3 that activates p53 to trigger its tumor-suppressive function [93], and lncRNAs CUPID1 and CUPID2, which are associated with the progression of breast cancer, modulating the DNA damage response [94]. Another hallmark of cancer, cellular metabolic disorders, can be regulated by lncRNAs. It has been shown that under energy stress, lncRNA NBR2 activates AMPK via direct binding, and absence of this lncRNA leads to changed metabolism and subsequent enhanced tumor cell proliferation [95]. MALAT1 has been extensively investigated for its function in tumorigenesis in NSCLC by promoting EMT and enhancing tumor progression and metastasis through the miR-124/STAT3 axis [96,97,98]. Additionally, HOTAIR is known to promote metastasis, such as in breast cancer, liver cancer, and pancreatic cancer, by activating the SMAD cascade signaling pathway, which induces EMT [99,100,101,102]. Cancer stemness is another important factor for tumor metastasis, since cancer cells with high stemness are able to survive and colonize other tissues. Several studies have shown that lncRNAs are involved in signaling pathways associated with stemness. In liver cancer cells, two lncRNAs, lncBRM and lncSox4, have been shown to participate in self-renewal through the YAP1 and the STAT3 pathways [103,104]. Altogether, lncRNAs have been shown to play a pivotal role in tumorigenesis, tumor progression, and metastasis.

### 3.3. LncRNAs in Immunity, Inflammation, and the TME

Although lncRNAs have been extensively described in a cancer and disease context, their role in immunity and inflammatory response is still not completely understood. The immune system consists of various cell types that mediate response to infections while maintaining tissue homeostasis [105,106]. Several studies have revealed that lncRNAs can influence proliferation, differentiation, and activation of immune cells such as monocytes, macrophages, dendritic cells, neutrophils, T cells, and B cells. For example, linc-Ccr2-5′AS is a Th2-associated lncRNA that regulates expression of Th2 genes in immune cells and has the ability to influence recruitment of Th2 cells to the lung [107]. Additionally, RNA sequencing analysis revealed a large number of lncRNAs specifically expressed in CD8+ and CD4+ T cells [108,109]. LncRNA Lethe was shown to be highly expressed in mouse embryonic fibroblasts, influencing NF-κB-dependent inflammatory response [110].

Within the tumor microenvironment, lncRNAs can act as modulators and communicators between immune cells and tumor cells. CASC2c was identified to regulate macrophage infiltration and polarization in glioblastoma by negatively regulating the expression of coagulation factor X [111]. Furthermore, lnc-EGFR was shown to promote differentiation of T reg cells, enhancing tumor progression of hepatocellular carcinoma [112].

### 3.4. LncRNAs in Macrophages in Lung Cancer

**Figure 3:**
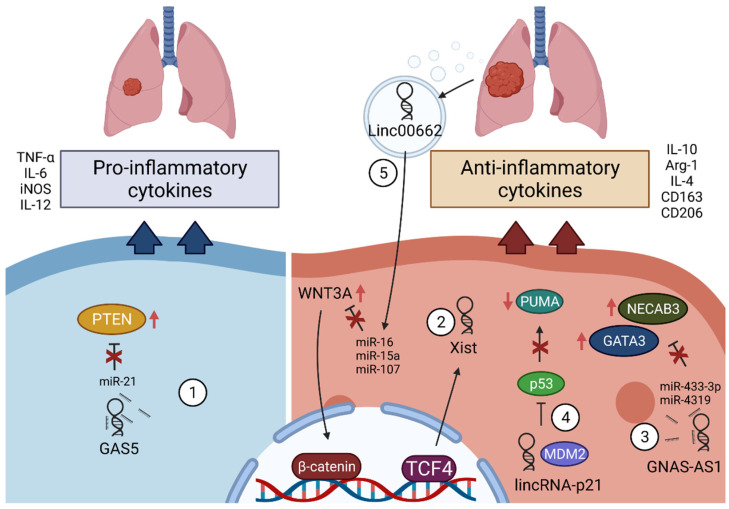
Influence of lncRNAs on modulation of TAMs of lung cancer. 1: GAS5 binds miR-21 in macrophages, upregulating PTEN and ultimately leading to an increase of proinflammatory cytokine and chemokine markers such as TNF-a, IL-6, iNOS, and IL-12. 2: Xist expression is upregulated in anti-inflammatory macrophages by transcription factor TCF4 and increases production of IL-10, CD163, and Arg-1. 3: GNAS-AS1 acts as a miRNA sponge binding miR-433-3p and miR-4319, leading to the upregulation of GATA3 and NECAB3, promoting CD206, IL-10, and Arg-1. 4: lincRNA-p21 interacts with MDM2 and inhibits the p53 complex, repressing transcription of the p53-associated gene PUMA, upregulating IL-10, IL-4, and Arg-1 and downregulating TNFa, IL-6, and iNOS. 5: Linc00662 is found in lung cancer patient plasma exosomes, lung cancer cells, and anti-inflammatory macrophages, suggesting that linc00662 is secreted into exosomes by cancer cells, activating the Wnt/ß-catenin signaling pathway in macrophages, training them to an anti-inflammatory M2 phenotype, and increasing CD163, IL-10, and Arg-1.

Although various lncRNAs seem to function in different cancer types and in all cell types of the TME, in the next chapters, we aim to focus on several examples of lncRNAs that, to our knowledge, are known to influence macrophage activation and polarization states and are associated specifically with lung cancer (Figure 3).

#### 3.4.1. GAS5

Growth arrest specific 5 (GAS5) is a long intergenic non-coding RNA (lincRNA) that has been shown to regulate the cell cycle in various systems [113], and its high expression inhibits tumor progression of several cancer types [114,115,116]. Therefore, it is widely accepted that GAS5 acts as a tumor suppressor. In NSCLC, GAS5 seems to be downregulated, whereas overexpression leads to decreased proliferation, enhanced apoptosis, and even reduced resistance to chemo- and radiotherapy [117,118,119]. Mechanistically, GAS5 acts as a sponge for several miRNA molecules such as miR-21 and miR-23a, resulting in upregulation of PTEN and increased sensitivity to cisplatin treatment [119]. Aside from its role in cancer cells, GAS5 also has been shown to have a regulatory function on immune response and on macrophages in the TME. High expression of GAS5 was found in human IFNɣ-stimulated M1 macrophages, again accompanied by elevated expression of PTEN and upregulation of inflammatory genes such as IL-12 and TNF-α [120]. Downregulation of GAS5 promotes macrophages toward an M2 phenotype, suppresses inflammation, and inhibits inflammatory cytokine release [120,121], supporting its role in macrophage polarization. Additionally, GAS5 overexpression in TAMs leads to decreased tumor cell migration, whereas GAS5 knockdown reverses this effect. PTEN knockdown in GAS5 overexpressed macrophages abolishes the effect on inflammatory cytokine expression and increased Arg-1 expression [120,122], whereas overexpression of PTEN inhibits M2 polarization [123], again highlighting the influence of this lncRNA on macrophages through the PTEN axis. This evidence suggests that GAS5 is an important regulator promoting the proinflammatory and anti-tumorigenic macrophage activation within the TME and that targeting GAS5 could serve as an option for lung cancer treatment.

Latest insights have shown that GAS5 can also regulate the metabolic state of cells by influencing energy production through the mitochondrial respiratory chain [91]. Since macrophage metabolism has also been shown to affect their activation state [76], further research is needed to evaluate the effect of GAS5-associated metabolic regulation on macrophage phenotype within the TME.

#### 3.4.2. Xist

The lncRNA X-inactive specific transcript (Xist) has been initially described in dosage compensation in mammalian cells by transcriptional silencing of the second X-chromosome in female cells [124]. More recently, functions beyond X-chromosome inactivation have been identified, including deregulation in several diseases and cancer, particularly lung cancer. Xist expression has been shown to be upregulated in NSCLC tissue and to increase cisplatin resistance in A549/DDP and H460/DDP lung adenocarcinoma cell lines [125,126] by sponging miR-144-3p and therefore leading to upregulation of the MDR1 and MRP1 chemoresistance genes. On the other hand, shRNA-mediated knockdown led to miR-144-3p–mediated downregulation of MDR1, MRP1, and reduced tumor-cell migration and invasion of lung cancer cells [126]. Emerging evidence also has shown Xist to be a regulator in macrophages, but its role is not yet completely clear. In some cases, high expression seems to be associated with inflammatory M1 polarization of macrophages such as in an osteoarthritis model and in IFNɣ/LPS-treated THP1 macrophages, whereas knockdown leads to a switch in polarization to a more M2 phenotype regulated via the mir-101-3p/KLF6/C/EBPa axis [127,128]. In the present study, downregulation of Xist in M1 macrophages led to higher proliferation and migration of breast and ovarian cancer cells. In lung cancer on the other hand, opposing studies have been shown which indicate that lncRNA Xist positively regulates M2 polarization of THP1 macrophages [129]. Furthermore, expression of Xist has not only been analyzed in the system of artificial cytokine-mediated polarization, but has also been shown to be increased in A549-conditioned macrophages, demonstrating the role of Xist in promotion of a pro-tumorigenic phenotype in TAMs associated with lung cancer. In this study, the transcription factor TCF-4 was found to be responsible for lncRNA-expression activation. Moreover, TCF-4 overexpression was able to restore downregulation of Xist-knockdown-associated downregulation of anti-inflammatory genes in macrophages such as IL-10, Arg-1, and CD163.

These findings support the characterization of Xist as an oncogenic factor in lung cancer not only from a tumor-cell site, but also in relation to macrophages in the TME. However, more studies are necessary to fully understand the underlying mechanisms and to clarify all aspects of Xist-regulation within macrophage activation.

#### 3.4.3. GNAS-AS1

GNAS Antisense RNA 1 (GNAS-AS1) is a non-coding RNA described only very recently that seems to be upregulated especially in cancerous tissue, such as NSCLC, promoting EMT and invasiveness of cancer cells [130,131], but its expression is also associated with M2 macrophage polarization [132]. Two studies have demonstrated that GNAS-AS1 overexpression in macrophages led to an anti-inflammatory phenotype by promoting CD206, IL-10, and Arg-1 expression [130,132]. Additionally, overexpression in macrophages led to higher proliferation, migration, and invasion of cancer cells. In tumor cells, GNAS-AS1 has been proposed to function via regulating the WNT/β-catenin pathway, whereas in macrophages, GNAS-AS1 seems to function as a miRNA-sponge. Both miR-433-3p and miR-4319 have been shown to bind to the lncRNA sequence, inhibiting degradation of GATA3 and NECAB3, which was already shown to have oncogenic functions [133].

Overall, these studies suggest an interesting role of GNAS-AS1 in tumor-promoting macrophages associated with NSCLC.

#### 3.4.4. LincRNA-p21

LincRNA-p21 appears to function as a component of the p53 signaling pathway by direct interaction with MDM2, a repressive complex of p53 leading to its degradation, thereby downregulating p53 target genes [134,135]. Additionally, lincRNA-p21 acts as a suppressor of translation by binding p53-target mRNAs [136]. P53 is a well-known tumor suppressor that is frequently mutated in cancer. Some studies have shown high expression of lincRNA-p21 in NSCLC [137], inhibiting cancer-cell apoptosis by decreasing expression of the p53-target gene PUMA. Recent evidence also suggests a role of p53 in the macrophage activation process [138]. A study using nutlin-3a, a putative p53 activator, was able to link activation of p53 with downregulation of anti-inflammatory genes, such as Arg-1, and a shift of the macrophage phenotype toward an M1-CD80^high^/CD206^low^ phenotype [138]. Another study confirmed these results by demonstrating an upregulation of lincRNA-p21 in TAMs, whereas knockdown facilitated macrophage polarization to M1 by upregulating TNF-α, IL-6, and iNOS, downregulating IL-10, IL-4, and Arg-1, and reducing tumor-cell proliferation and migration [139]. Moreover, some data suggests that lincRNA-p21 expression is induced by hypoxia [140,141] and high expression in NSCLC was connected with poor survival and an increased number of blood vessels accompanied by upregulation of angiogenesis-related genes, such as VEGFA, MMP2, FGF2, or PDGFB [140]. Moreover, those studies showed that lincRNA-p21 might serve as a prognostic marker of relapse after a resection of NSCLC.

Taken together, linc-p21 seems to be an important factor associated with enhanced lung tumor development not only by inhibition of p53-mediated apoptosis and promotion of VEGF-associated angiogenesis in lung cancer, but also by enhancing the phenotypic switch of TAMs in the TME toward a pro-tumorigenic M2 phenotype, further escalating tumor progression.

#### 3.4.5. Linc00662

In human lung carcinoma, elevated levels of linc00662 were detected in cancer cells and as well as plasma exosomes of NSCLC patients and have been correlated with poor survival [142,143,144]. In cancer cells, linc00662 has been shown to upregulate WNT3 through binding miR-15a/16/107, activating the WNT/β-catenin signaling pathway, increasing proliferation and migration abilities of tumor cells in vitro, and promoting tumor growth in vivo [23]. Furthermore, it was shown that β-catenin protein levels were higher in macrophages treated with conditioned media of linc00662 cancer cells compared to conditioned media of control or linc00662-silenced cells. These findings together with the presence of linc00662 in exosomes in lung cancer patients suggest a regulatory role of this lncRNA in a paracrine manner. Moreover, activation of the WNT/β-catenin pathway in overexpressed-CM-treated macrophages was associated with the inhibition of IL-12, iNOS, and TNF-α and increased M2 macrophage markers such as CD163, IL-10, and Arg-1. These findings are consistent with those of previous studies that identified WNT/β-catenin signaling as a crucial pathway in reprogramming of TAMs [76]. Overall, these data suggest a tumor-promotive role of linc00662 in cancer, while simultaneously being secreted into the TME through exosomes, which promotes macrophages towards a M2 phenotype.

Although the underlying mechanisms still need to be further elucidated, the available literature suggests that linc00662 could serve as a biomarker in lung cancer and that targeting this lncRNA could improve patient survival by the reeducation of TAMs to an anti-tumorigenic phenotype.

### 3.5. Future Prospects of lncRNAs in Tumor Management and Therapy

Recent studies reveal the potential of RNA therapeutics as a new strategy to target diseases. Considering the diverse roles of lncRNAs, RNA-based therapies may represent promising approaches as therapeutic options, e.g., for diseases with a poor outcome with available treatment options. Until now, there are several approaches to target ncRNAs in vivo, such as small interfering RNAs (siRNAs), antisense oligonucleotides (AO), e.g., shRNAs or LNA GapmeRs triggering RNase H-mediated RNA degradation, as well as CRISPR/Cas9 genome editing. Some of these methods are tested in clinical trials for diverse pathologies, such as neurological disorders or cardiovascular complications [145,146,147]. For example, the AO-based therapeutic Nusinersen was approved by the FDA to treat spinal muscular atrophy, degrading incorrect mRNA-splice forms of the survival motor neuron (SMN) gene [145,148]. The LNA GapmeR-based antisense oligonucleotide MRG-110 targets miR-92a-3p and is currently being tested in phase 2 clinical trials as an anti-inflammatory drug [149,150]. Additionally, for cancer, several clinical trials are ongoing, e.g., targeting G12D-mutated KRAS mRNA in advanced pancreatic cancer, targeting STAT3 mRNA in metastatic NSCLC, or HSP27 mRNA in lung cancer, metastatic bladder cancer, or prostate cancer [151]. Additionally, non-coding RNAs as targets are tested in cancer clinical trials; however, these are mainly focused on miRNAs, while a search for lncRNA clinical trials by ClinicalTrials.gov are often limited and associated with a search for biomarkers (Table 1). Expression modulation of some lncRNAs has already been investigated in several mouse models (Table 2); however, there are many promising targets involved in different processes of tumor progression that would be worth investigating in more detail in vivo as well as in future clinical trials. A small selection of these lncRNAs can be seen in Table 3.

## 4. Summary/Conclusions

As a result of late diagnosis and cancer biology, lung cancer still has a devastating 5-year survival of around 15%. The latest developments in targeted therapy and immunotherapy have already led to advances in treating patients, but only a small subset of patients within a large cohort responds favorably to the treatment and relapse may occur [167]. Additionally, increasing resistance to classic chemotherapeutics demonstrates the need of new target strategies.

Recently, regulatory functions of lncRNAs were appreciated in several cancer types as well as lung cancer in particular. This review highlights their role in immune cells and modulation of TAMs phenotype within the TME. However, the properties of ncRNAs are thought to be very heterogeneous in their function, which makes the field quite complex and the specific role in lung tumor progression difficult to understand. An increasing number of lncRNAs that enhance either the inflammatory M1 or anti-inflammatory M2 activation state of macrophages have been described, which is why some lncRNAs act as tumor suppressors and others rather as oncogenes. Additionally, the role of one lncRNA seems to differ among cancer types, as exemplified by the lncRNA Xist, in which high expression in macrophages enhances proliferation and migration of breast and ovarian cancer, whereas macrophages associated with lung cancer have shown the opposite effect.

Considering the expression profile in cancerous tissue, immune cells, or plasma of patients, lncRNAs could potentially become important as indicators of disease, minimally invasive biomarkers, and even expand to RNA therapeutics, serving as promising new targets in lung cancer through modulation of the TME. Moreover, since various lncRNAs seem to modulate chemoresistance, their combination with conventional treatments seems likely. Nevertheless, more studies are needed to decipher the underlying molecular pathways in greater detail. Additionally, effective knockdown or overexpression strategies for lncRNAs need to be explored. Presently, RNA therapy is restricted by competent delivery approaches since siRNAs have been shown to not be as effective in vivo as in in vitro studies, as well as have some off-target effects. Additionally, the complex lung structure is quite difficult to penetrate for siRNA-mediated therapeutics. Nanoparticle or viral vector-based delivery of antisense oligonucleotides could be a way to use RNAi-based therapies in vivo, although these techniques need to be further studied to assess their efficacy, dose control, specificity, and accuracy for achieving the desired therapeutic effect. LncRNA-targeted therapy is just getting started, but has the potential to become a vast therapeutic tool for cancer treatment.

Long considered to be “junk RNA”, lncRNAs are now perceived as crucial players in regulating immunological signaling pathways, including macrophage polarization, macrophage-tumor cell-crosstalk, and lung cancer progression. A deeper understanding of lncRNAs and their tumor-suppressive or oncogenic function is crucial for their future use as biomarkers and novel therapeutic tools regulating the TME in lung cancer.

## Figures and Tables

**Table 1:** Clinical trials associated with lncRNAs and cancer (ClincalTrials.gov).

lncRNA	NCT Number	Clinical Trial	Cancer Type	Phase
HOTAIR	NCT03469544	Biomarker	Thyroid cancer	Recruiting
THRIL, PACER	NCT03057171	*H. pylori*-controlled lncRNA	Stomach cancer	unknown
H19	NCT04767750	Regulation of IGF-1R Expression	Hepatocellular carcinoma	Recruiting
MFI2-AS1	NCT04946266	Validation of prognostic value	Kidney cancer	Not yet recruiting
Serum exosomal ncRNAs	NCT03830619	Potential biomarker for diagnosis	Lung cancer	Unknown
Xist	NCT04288739	Immunophenotyping	Acute myeloid leukemia	Not yet recruiting

**Table 2:** Approaches of lncRNA targeting for cancer treatment in vivo.

lncRNA	Mouse Model	Result	Therapy Approach	Reference
MALAT 1	Lung cancer, Breast cancer	Reduced metastasis	AO	[152,153]
DANCR	Breast cancer	Reduced tumor growth	siRNA nanoparticles	[154]
AC104041.1	HNSCC	Inhibition of tumor growth	AO	[155]
LINC01296	NSCLC	Reduced tumor mass	siRNA	[156]
HOTAIR	Ovarian cancer, Breast cancer	Reduced tumor formation, improved survival	AO	[157]
NEAT1	Multiple myeloma	Antitumor activity	AO	[158]
MALAT1	Multiple myeloma	Antitumor activity, cytotoxic effect	AO + Bortezomib	[159]

**Table 3:** Selection of possible lncRNA targets for future lung cancer therapy.

lncRNA	Expression in LC	Involved Process	Possible Benefit	Reference
HOTAIR H19 SNHG1	High	Biomarker in Sputum	Early and non-invasive diagnosis	[160]
LOC146880 Xist	High	Biomarker in Serum	Early diagnosis	[161]
H19	High	Proliferation, Cisplatin resistance	Inhibition of tumor growth, improvement of chemotherapy	[162,163]
HOTAIR	High	Migration, Cisplatin resistance	Reduced metastasis, improvement of chemotherapy	[164]
BANCR	Low	Migration, radioresistance	Reduced metastasis, improvement of radiotherapy	[165]
ANRIL	High	Proliferation, radioresistance	Reduced tumor growth, improvement of radiotherapy	[166]
